# Independent component analysis reveals 49 independently modulated gene sets within the global transcriptional regulatory architecture of multidrug-resistant *Acinetobacter baumannii*

**DOI:** 10.1128/msystems.00606-23

**Published:** 2024-01-08

**Authors:** Nitasha D. Menon, Saugat Poudel, Anand V. Sastry, Kevin Rychel, Richard Szubin, Nicholas Dillon, Hannah Tsunemoto, Yujiro Hirose, Bipin G. Nair, Geetha B. Kumar, Bernhard O. Palsson, Victor Nizet

**Affiliations:** 1 School of Biotechnology, Amrita Vishwa Vidyapeetham, Amritapuri, Kerala, India; 2 Division of Host-Microbe Systems and Therapeutics, Department of Pediatrics, University of California, San Diego, La Jolla, California, USA; 3 Department of Bioengineering, University of California, San Diego, La Jolla, California, USA; 4 Department of Biological Sciences, University of Texas at Dallas, Dallas, Texas, USA; 5 Division of Biological Sciences, University of California, San Diego, La Jolla, California, USA; 6 Department of Microbiology, Graduate School of Dentistry, Osaka University, Suita, Osaka, Japan; 7 Skaggs School of Pharmacy and Pharmaceutical Sciences, University of California, San Diego, La Jolla, California, USA; Rice University, Houston, Texas, USA

**Keywords:** *Acinetobacter baumannii*, transcriptional regulation, iModulon

## Abstract

*Acinetobacter baumannii* causes severe infections in humans, resists multiple antibiotics, and survives in stressful environmental conditions due to modulations of its complex transcriptional regulatory network (TRN). Unfortunately, our global understanding of the TRN in this emerging opportunistic pathogen is limited. Here, we apply independent component analysis, an unsupervised machine learning method, to a compendium of 139 RNA-seq data sets of three multidrug-resistant *A. baumannii* international clonal complex I strains (AB5075, AYE, and AB0057). This analysis allows us to define 49 independently modulated gene sets, which we call iModulons. Analysis of the identified *A. baumannii* iModulons reveals validating parallels to previously defined biological operons/regulons and provides a framework for defining unknown regulons. By utilizing the iModulons, we uncover potential mechanisms for a RpoS-independent general stress response, define global stress-virulence trade-offs, and identify conditions that may induce plasmid-borne multidrug resistance. The iModulons provide a model of the TRN that emphasizes the importance of transcriptional regulation of virulence phenotypes in *A. baumannii*. Furthermore, they suggest the possibility of future interventions to guide gene expression toward diminished pathogenic potential.

**IMPORTANCE:**

The rise in hospital outbreaks of multidrug-resistant *Acinetobacter baumannii* infections underscores the urgent need for alternatives to traditional broad-spectrum antibiotic therapies. The success of *A. baumannii* as a significant nosocomial pathogen is largely attributed to its ability to resist antibiotics and survive environmental stressors. However, there is limited literature available on the global, complex regulatory circuitry that shapes these phenotypes. Computational tools that can assist in the elucidation of *A. baumannii*’s transcriptional regulatory network architecture can provide much-needed context for a comprehensive understanding of pathogenesis and virulence, as well as for the development of targeted therapies that modulate these pathways.

## INTRODUCTION

Multidrug-resistant (MDR) *Acinetobacter baumannii* has rapidly emerged as a critical threat to global public health since its notoriety as a pathogen in servicemen in the Iraq and Afghanistan wars (1) to more recent intensive care unit outbreaks during the COVID-19 pandemic (2–4). An aerobic, nonmotile, pleomorphic Gram-negative coccobacillus, *A. baumannii* causes healthcare-associated infections including ventilator-associated pneumonia (VAP), catheter-associated urinary tract infections (CAUTI), septicemia, and wound infections (5–8). Attributable healthcare costs for *A. baumannii* infections in the United States were $281 million in 2017 (9), a figure continuing to rise. The resiliency of the pathogen in hospital environments reflects its tolerance of stressful environments and resistance to most of the available antibiotic regimens. In particular, carbapenem-resistant *A. baumannii* (CRAB) strains are atop the list of priority pathogens for new antibiotic research and development articulated by the US Centers for Disease Control and Prevention and World Health Organization (9, 10). Increasing reports of fulminant community-acquired *A. baumannii* in immunocompromised individuals point to shifting trends in transmission and virulence potential, emphasizing the importance of fundamental pathogen biology knowledge to guide alternative treatment strategies (11–13).

Elucidation of the intricate molecular processes that underpin *A. baumannii* survival and spread in host tissues lags behind other longer-studied nosocomial pathogens. Prior studies demonstrate changes in the external environment lead to complex fluctuations in bacterial gene expression profiles that allow a given microbe to adapt and promote advantageous survival phenotypes (14). Over the past decade, increasing attention has been paid to the role of *A. baumannii* transcriptional regulators in virulence, pathogenesis, survival, and antibiotic resistance. These pioneering studies have focused principally on the realms of comparative genomics (15), whole-genome screens to identify regulators with roles in pathogenesis (16, 17), and functional investigations into individual regulators, but leave a gap in elucidation of a global *A. baumannii* transcriptional regulatory network (TRN). An unbiased global analysis of the *A. baumannii* TRN could answer questions about how this pathogen can persist outside of the host and cause severe infection within it. With that objective in mind, we turned to the methodology of independent component analysis (ICA).

ICA is an unsupervised machine-learning algorithm that extracts independent source signals from a mixture (18). When applied to a compendium of RNA-seq data from a single organism, ICA extracts independently modulated sets of genes that we have termed iModulons (19). The genes in each iModulon represent a set whose expression levels are modulated independently throughout the data set, likely by the same underlying source signal. The source signal may represent a single regulator, combinations of multiple regulators, or genomic features with coordinated gene expression such as prophages and pathogenicity islands (20, 21). iModulons can also capture strain differences, either between related strains or from genetic manipulations. In addition, ICA calculates the activity of each iModulon in every input sample, providing insight into the dynamic behavior of each gene set. Prior ICA analyses have been successful in defining biologically relevant iModulons in a number of prokaryotic organisms including *Escherichia coli* (19, 22), *Staphylococcus aureus* (20), *Bacillus subtilis* (23), *Sulfolobus acidocaldarius* (24), *Pseudomonas aeruginosa* (25, 26), *Mycobacterium tuberculosis* (27)*, Pseudomonas putida* (28), and *Streptococcus pyogenes* (29).

In this study, we scraped the Sequence Read Archive (SRA) for all publicly available RNA-seq profiles of *A. baumannii* clonal complex 1 (CC1) and processed and quality controlled them to generate a large compendium of data on which to apply ICA. We also generated 74 expression profiles ourselves. This resulted in 139 high-quality RNA-seq samples from the *A. baumannii* strains AB5075, AYE, and AB5007 (30–32). ICA extracted 49 robust iModulons from this data set. We showcase how analysis of the *A. baumannii* iModulons can (i) identify genes that may be modulated together in an organism in the absence of prior TRN knowledge (ii), provide insight into a potential RpoS-independent general stress response (iii), define a possible global regulatory stress-virulence trade-off dynamic, and (iv) highlight growth conditions that may induce plasmid-borne antibiotic resistance. This data set is provided in its entirety to the larger scientific community on iModulonDB.org, where interested users can search the identified *A. baumannii* iModulons, and view or interrogate them through interactive dashboards (33). We hope that this framework can help inspire additional research to understand the virulence-centric regulatory networks of this high- priority emerging MDR pathogen with an eye toward potential new antibiotic or anti-virulence therapeutics.

## RESULTS

### Independent component analysis extracts 49 independently modulated gene sets (iModulons) of *A. baumannii*

**Fig 1:**
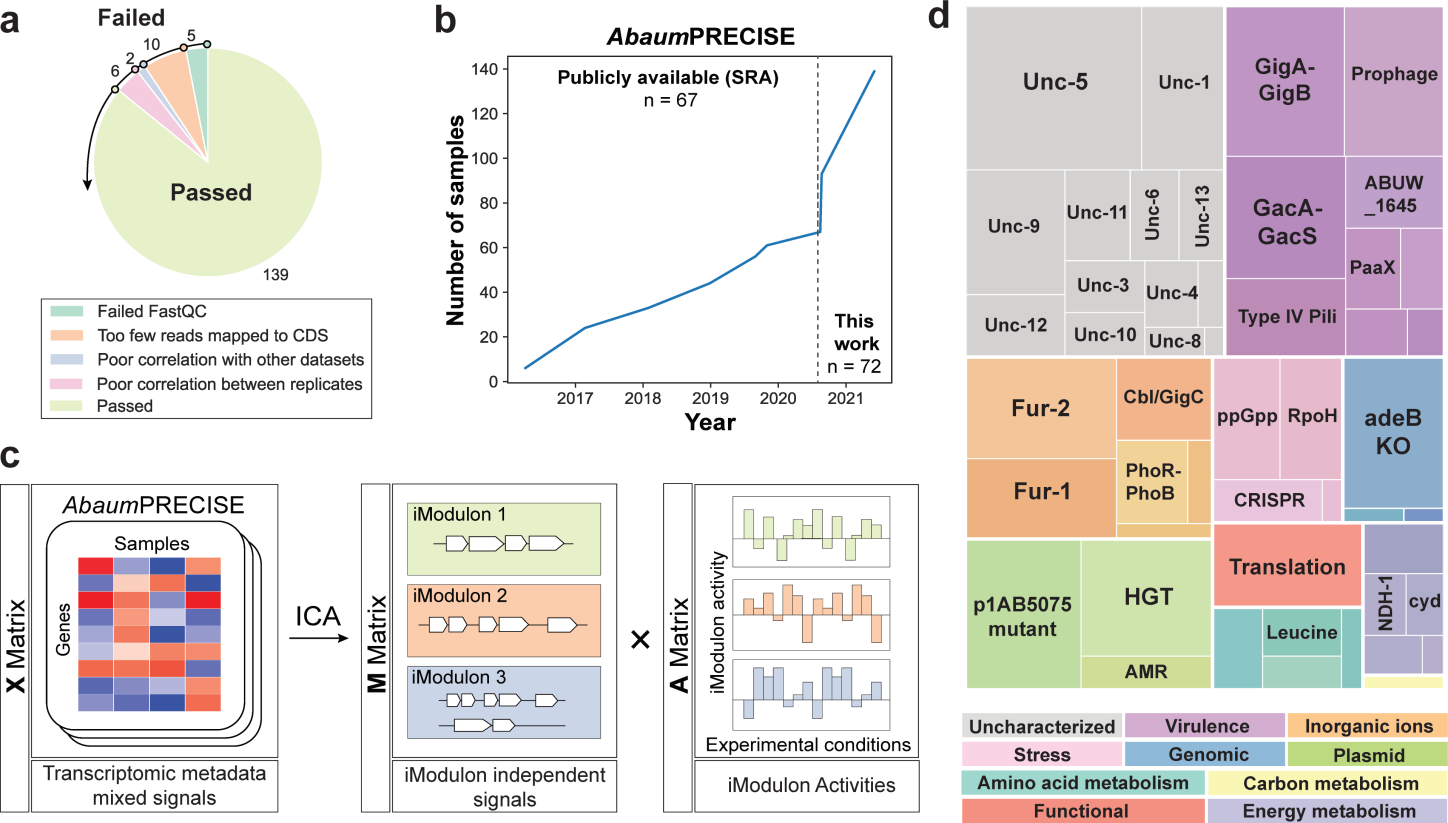
Curation of *Abaum*PRECISE and computation of *A. baumannii* iModulons. (**a**) 162 available RNA-seq data sets (collected from SRA and generated in this study) for *A. baumannii* strains AB5075, AYE, and AB0057 went through a QC/QA pipeline, resulting in the final 139 samples that made up *Abaum*PRECISE. (**b**) *Abaum*PRECISE data collection over time. (**c**) General pymodulon workflow used to generate *A. baumannii* AB5075 iModulons. Independent component analysis was run on *Abaum*PRECISE RNA-seq metadata (**X** matrix) defining distinct independently modulated signals from gene sets or iModulons (**M**) as well as the activity of these iModulons in the defined experimental conditions of each RNA-seq sample (A). (**d**) Treemap of *A. baumannii*’s 49 independently modulated gene sets or iModulons. Each box represents a single iModulon while box size refers to the number of genes within the iModulon. Complete information on each iModulon can be found on the interactive dashboard on iModulonDB.org.

We constructed “*Acinetobacter baumannii* Precision RNA-seq Expression Compendium for Independent Signal Extraction (*Abaum*PRECISE)” by collecting all publicly available RNA-seq data for *A. baumannii* CC1 strains AB5075, AYE, and AB0057 from SRA published from 2016 to mid-2020. Even though there are abundant publicly available RNA-seq experiments for other *A. baumannii* strains (like ATCC 17978), we limited our metadata to these three commonly studied, multidrug-resistant strains to limit the fraction of iModulons that capture genetic variation instead of true transcriptional regulatory units. An additional 74 expression profiles generated in our laboratory (‘in-lab”) were incorporated into an initial compendium of 162 RNA-seq profiles from both wild-type and mutant AB5075 strains grown in a variety of conditions. In total, 139 of these data sets passed an established QC/QA pipeline (34) and were curated as *Abaum*PRECISE (Fig. 1a). *Abaum*PRECISE thus includes a total of 58 unique conditions collected over 5 years (Fig. 1b). The majority of these represent snapshots of bacterial responses to clinically relevant scenarios during pathogenesis such as changes in growth parameters, antibiotic treatment, or isogenic mutants in virulence-associated genes. The details of these samples as well as their normalized log TPM values are listed in Data Sets S1 and S2.

As detailed in previous work (19), when an **X** matrix consisting of RNA-seq metadata is used as the input for ICA, the resulting in **M** (iModulon) and **A** (activity) matrices provide information on the sets of genes that are modulated independently (presented by the weights in a column in **M**) and the activities of these gene sets across the experimental conditions (presented by the entries in a row in **A**), respectively (Fig. 1c). Complete X, M, and A matrices are listed in Data Sets S2, S3, and S4. ICA was run on *Abaum*PRECISE to decompose the expression metadata into 49 independently modulated gene sets or iModulons (Fig. 1d; Data Set S5). In total, these iModulons explain 56.6% of the total variance in the input RNA-seq data. An interactive dashboard on these iModulons and their activities across *Abaum*PRECISE conditions is available at iModulonDB.org.

Since *A. baumannii* is less well defined at the gene regulation level compared to other bacterial pathogens (33), iModulon characterization could not be accomplished by simply searching for similarities to known regulons. Each iModulon was first characterized and annotated based on functional enrichment of iModulon gene contents to a manually curated known TRN based on RegPrecise data and prior literature. We validated potential prophage iModulons by comparing iModulon gene sets to PHASTer-defined prophage regions in the same manner. Characterization was furthered by manual annotation of the gene sets and their comparison to iModulons in other Gram-negative bacteria: *E. coli* (35) and *P. aeruginosa* (25). In this manner, 36 out of the 49 iModulons were annotated with a regulator and/or function; each iModulon was also generally categorized based on function.

The categories with the largest number of iModulons were those associated with virulence (*n* = 9) and inorganic ions (*n* = 6). The importance of two-component signal transduction systems (TCS) for *A. baumannii* was evident with four iModulons capturing the effects of PhoR-PhoB, GigA-GigB, GacA-GacS, and KpdD-KpdE. In all, 13 iModulons remained uncharacterized, owing largely to the limited knowledge of the complete *A. baumannii* TRN. These iModulons may represent gene sets under the control of unknown regulators and open an avenue to define new gene-regulator relationships.

In the sections that follow, we will illustrate how ICA modeling of transcriptomic data can provide accurate biological representations of the complex *A. baumannii* gene regulatory system thus providing a framework for discovery. Through specific examples, we highlight how *Abaum*PRECISE iModulons suggest possible mechanisms underlying *A. baumannii* phenotypes related to virulence, survival, and antibiotic resistance.

### Validation that iModulons capture independently modulated gene sets

**Fig 2:**
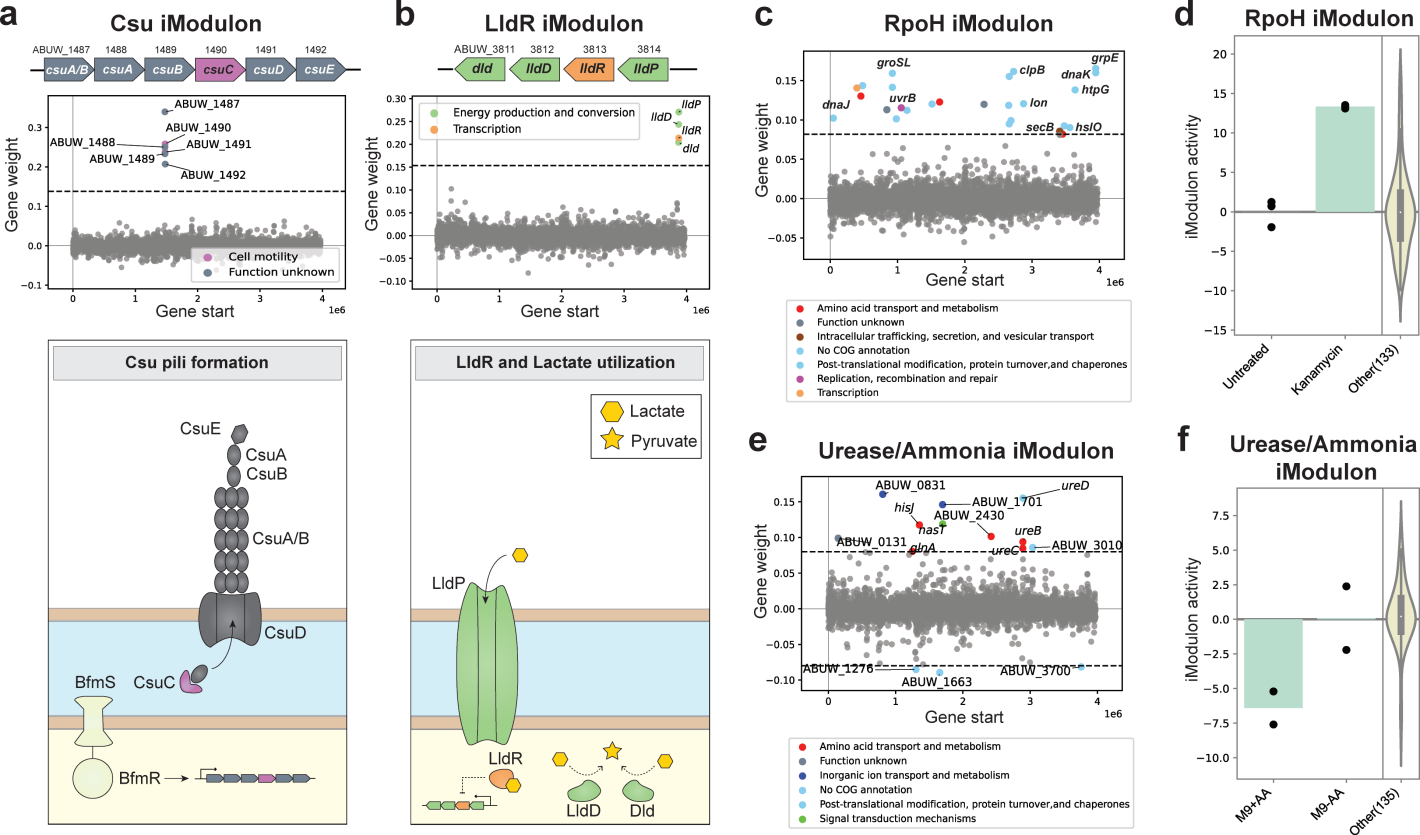
Validation of computed iModulons and their potential for biologically relevant discoveries. Gene weight plots for the (**a**) Csu and (**b**) LldR iModulons completely capture the Csu pili operon under the BfmR-BfmS two-component system regulation and the LldR operon/regulon involved in lactate utilization and images of their physical embodiment. (**c**) The gene weight plot for the RpoH iModulon consists of multiple chaperonins, chaperone proteins, and heat shock proteins. (**d**) RpoH activity increased when bacteria were treated with kanamycin, a known inducer of the heat shock response. (**e**) Gene weight plot for the Urease/Ammonia iModulon highlights genes involved in urea conversion to ammonia and ammonia import. (**f**) AB5075 grown in minimal M9 media supplemented with amino acids results in decreased Urease/Ammonia iModulon activity compared to bacteria grown in unsupplemented M9 media. Bars represent mean values of replicate values (represented by points) within each condition and violin plot represents the range of activity across other conditions within *Abaum*PRECISE.

iModulons captured known regulons/operons in *A. baumannii* using expression data alone. For example, the Csu pili operon consisting of six *csu* genes was captured as a single iModulon annotated as the Csu iModulon (Fig. 2a). Prior studies reported that this operon is regulated by the BfmR-BfmS two-component system which plays a variety of roles in antibiotic resistance, virulence, and stress response (36, 37). This pattern held true even for regulons/operons that did not have RNA-seq samples collected under growth conditions that would explicitly select for them, as seen in the case of the LldR regulon. This regulon encodes genes involved in the control of lactate utilization. The LldR regulon was extracted as a single iModulon even though *Abaum*PRECISE did not include any experimental conditions involving lactic acid supplementation to the growth media (Fig. 2b).

### iModulons help define new regulons and nutritional prioritization

*Abaum*PRECISE iModulons yielded novel information on regulator effectors and their activities under certain conditions allowing better characterization of the TRN. For example, although the RpoH regulon was not previously defined in *A. baumannii*, we could designate an iModulon consisting of multiple chaperonins, chaperone proteins, and heat shock proteins as the RpoH iModulon because its gene weights were correlated with the RpoH iModulons of *E. coli* and *P. aeruginosa* when genes were matched based on bidirectional best hits from BLAST (25, 35, 38) (Fig. 2c; Fig S1). Correspondingly, the *A. baumannii* RpoH iModulon activity increased when bacteria were treated with kanamycin, a known inducer of the heat shock response (39, 40) (Fig. 2d), consistent with its expected function. Another easily interpretable iModulon is the Urease/Ammonia iModulon which contains genes involved in urea conversion to ammonia and ammonia import (Fig. 2e). When bacteria were grown in minimal M9 media, amino acid supplementation resulted in decreased Urease/Ammonia iModulon activity (Fig. 2f), suggesting that *A. baumannii* prefers amino acids over urea as a nitrogen source under the tested conditions.

### *A. baumannii* phase variation and the ABUW_1645 iModulon

**Fig 3:**
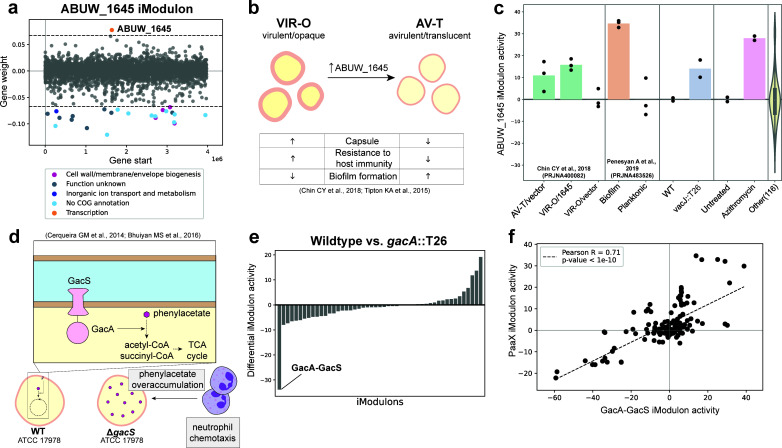
The ABUW_1645 and GacA-GacS iModulons. (**a**) Gene weight plot of ABUW_1645 iModulon. (**b**) Prior literature reports that the expression of ABUW_1645 mediates the phase variation switch from VIR-O to AV-T populations and describes the phenotypes of each. (**c**) ABUW_1645 iModulon activities in (i) VIR-O, AV-T, and VIR-O overexpressing ABUW_1645 populations; (ii) biofilm versus planktonic growth; (iii) wild type versus *vacJ* transposon mutant; and (iv) untreated versus azithromycin-treated AB5075. Bars represent mean values of replicate values (represented by points) within each condition and violin plot represents the range of activity across other conditions within *Abaum*PRECISE. (**d**) The GacA-GacS two-component system is associated with the phenylacetic acid (Paa) catabolism pathway. A previous study suggested that ATCC 17978 mutants that lacked a functional GacA-GacS system had phenylacetate overaccumulation which led to neutrophil chemotaxis to the site of infection and increased bacterial clearance in the host. (**e**) Differential iModulon activity (DIMA) analysis of all iModulons in a *gacA* transposon mutant compared to wild-type AB5075. The GacA-GacS iModulon was defined as the iModulon with the lowest DIMA. (**f**) iModulon activity plot of GacA-GacS and Paax iModulons across all conditions within *Abaum*PRECISE highlights the positive correlation between the activities of these two iModulons.

The ABUW_1645 iModulon consists of 23 genes with negative gene weights and only one gene, ABUW_1654, with a positive gene weight (Fig. 3a). ABUW_1645 is a global regulator of the phase variation switch between virulent, opaque (VIR-O) and avirulent, translucent colony (AV-T) type populations in AB5075 (41, 42). Overexpression of ABUW_1645 induces the phase switch from a virulent, opaque colony type (VIR-O) phenotype associated with capsule production and increased survival in the host to an avirulent, translucent colony type (AV-T) phenotype that, despite producing increased biofilm, has limited pathogenicity (Fig. 3b). Activity analysis of the ABUW_1645 iModulon provided expected and new insights (Fig. 3c). As expected, the ABUW_1645 iModulon has increased activity in VIR-O overexpressing ABUW_1645 and in AV-T compared to VIR-O populations of AB5075. Although multiple iModulons either contained ABUW_1645 or were enriched to the ABUW_1645 regulon, the ABUW_1645 iModulon characterization was based on the observations that only this iModulon contained ABUW_1645, was enriched for regulator ABUW_1645, and had differential iModulon activity in VIR-O/AV-T phase switching data. Increased ABUW_1645 iModulon activity in biofilm growth is likewise consistent with prior experimental observations (43). Surprisingly, we found that treatment with azithromycin increased ABUW_1645 iModulon activity, suggesting that this translation-inhibiting antibiotic could dampen *A. baumannii* virulence *via* this pathway. Lastly, ABUW_1645 iModulon activity also increased in a transposon mutant disrupting the *A. baumannii vacJ* gene compared to wild-type AB5075; *vacJ* homolog in *P. aeruginosa* encodes a key stabilizer of outer membrane lipid asymmetry; and loss-of-function mutants have decreased virulence in the host (44). This observation suggests that *vacJ* inhibitors may hinder virulence through ABUW_1645-mediated phase variation in addition to outer membrane structure.

### Phenylacetic acid (Paa) catabolism and the GacA-GacS iModulon

The GacA-GacS two-component system is a global virulence regulator linked to increased virulence *via* the phenylacetic acid (Paa) catabolic pathway. An isogenic *gacS* mutant of *A. baumannii* ATCC 17978 was previously reported to stimulate increased neutrophil chemotaxis to the site of infection due to an overaccumulation of phenylacetate, a neutrophil chemoattractant (45, 46) (Fig. 3d). Differential iModulon activity analysis comparing *gacA*::T26 to wild-type AB5075 highlighted decreased activity in an uncharacterized iModulon that we subsequently designated the GacA-GacS iModulon (Fig. 3e). Although this iModulon does not capture the expected Paa pathway operon, we observed high positive correlation between the activities of the GacA-GacS and PaaX iModulons, supporting this manual annotation (Fig. 3f; Fig S2).

### RpoS-independent stress responses and the ABUW_1645 and GacA-GacS iModulons

**Fig 4:**
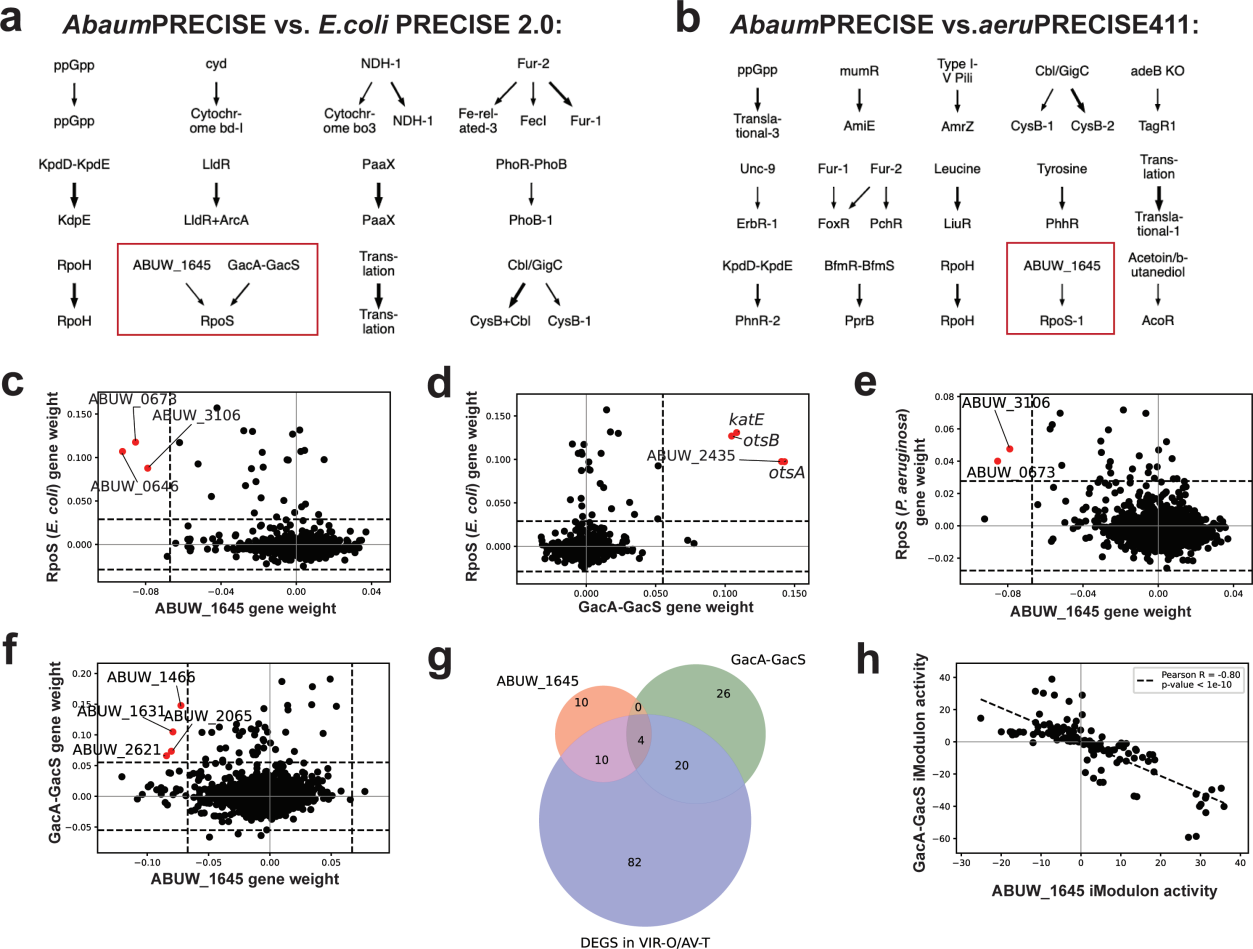
ABUW_1645 and GacA-GacS iModulons may represent an RpoS-independent general stress response. Correlating iModulons from *Abaum*PRECISE and (**a**) *E.coli* PRECISE 2.0 and (**b**) *aeru*PRECISE411 based on bidirectional best hit (BBH) orthology of iModulon genes. The top iModulons are from *A. baumannii* and the bottom iModulons are from either *E. coli* or *P. aeruginosa*, respectively. The thickness of the arrow represents a greater correlation. Comparison of gene weights of (**c**) *A. baumannii* ABUW_1645 iModulon with *E. coli* RpoS iModulon, (**d**) *A. baumannii* GacA-GacS iModulon with *E. coli* RpoS iModulon, and (**e**) *A. baumannii* ABUW_1645 iModulon with *P. aeruginosa* RpoS iModulon showcases The similarities between the iModulons. The red data points in comparative gene weight plots highlight genes that are common to both compared iModulons. (**f**) Comparative gene weight plots between ABUW_1645 and GacA-GacS iModulons show that they have four genes in common, all of which are hypothetical proteins with unknown functions. (**g**) Venn diagram of comparing the genes within the ABUW_1645 and GacA-GacS iModulons and those differentially expressed between VIR-O and AV-T populations. (**h**) The plot of iModulon activities of ABUW_1645 and GacA-GacS across all conditions within *Abaum*PRECISE highlights a strong negative correlation between their activities.

RpoS is a well-characterized general stress response sigma factor found across bacterial species and often ancillary to the diphasic lifestyles of many Gram-negative pathogens within and outside of the host (47). Surprisingly, AB5075 does not harbor an *rpoS* homolog (37, 48), but a comparison of the gene weights within the ABUW_1645 and GacA-GacS iModulons reveals similarity to RpoS iModulons in *E.coli* and *P. aeruginosa* (Fig. 4a through e). Curiously, only four genes were found in common in both ABUW_1645 and GacA-GacS, all of which are annotated as hypothetical proteins with unknown function (Fig. 4f). DEGs between VIR-O and AV-T published previously (41) are accounted for by both the ABUW_1645 and GacA-GacS iModulons (Fig. 4g). This evidence suggests that ABUW_1645 and GacA-GacS iModulons capture the activities of regulators that may induce opposing shifts across the virulence spectrum, supported further by the high negative correlation between ABUW_1645 and GacA-GacS activities across our metadata (Fig. 4h). Based on these observations, we propose that a virulence-limiting ABUW_1645 and virulence-inducing GacA-GacS dichotomy may play opposing roles in regulating phase variation switching and represent an *A. baumannii* RpoS-independent general stress response.

### Clustered activity of five iModulons captures potential global regulatory stress-virulence tradeoffs

**Fig 5:**
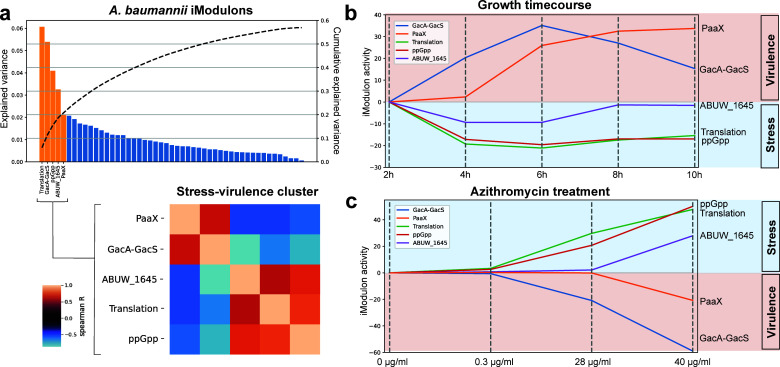
Global regulatory stress versus virulence trade-offs in *A. baumannii*. (**a**) Explained variance of the 49 *A*. *baumannii* iModulons. Each bar represents the explained variance of each independent iModulon with values corresponding to the left Y-axis while cumulative explained variance is represented as the black dotted line in association with the right Y-axis. iModulon activity clustering analysis clusters the top five iModulons as members of the stress-virulence cluster. Stress-virulence cluster activity (**b**) over the growth time course of AB5075 grown in RPMI +10% LB and (**c**) treatment with increasing concentrations of azithromycin. Sampling for time course transcriptomic analysis was done every 2 hours from 2 to 10 hours and normalized to 2-hour time point while azithromycin treatment was done at 0.3, 28, and 40 µg/mL and normalized to untreated (0 µg/m) control.

Analysis of variance explained by *Abaum*PRECISE iModulons can provide information on possible global regulators. Clustering of the iModulon activities across an array of experimental conditions yielded a potential global regulatory cluster consisting of the five iModulons with the highest explained variance: Translation, GacA-GacS, ppGpp, ABUW_1645, and PaaX (Fig. 5a; Fig S3). As we associated ABUW_1645 and GacA-GacS with the general stress response and phase switching between virulent and avirulent phenotypes, we defined this cluster as a global regulatory stress-virulence cluster. During initial characterization, the Translation and ppGpp iModulons were classified as two putative Translation iModulons as they each contained numerous genes encoding ribosomal proteins (Fig S4a and b). However, the gene weights of these two iModulons had a high correlation to those of the *E. coli* Translation and ppGpp iModulons, prompting the re-annotation of the iModulons (Fig S4c). ppGpp regulates induction of the stringent response under stressful, nutrient-limited conditions in many pathogens, and recent studies in *A. baumannii* show implications for virulence (49, 50). Stress, or avirulent, phenotypes may be induced by the ppGpp, Translation, and ABUW_1645 iModulons, while virulence phenotypes may be induced by the GacA-GacS and PaaX iModulons.

This divergence in the activities of this cluster is clearly portrayed in experiments observing growth over time (Fig. 5b). When normalized to activities at 2 hours, during peak logarithmic growth (4–6 hours), the stress iModulons have decreased activity while virulence iModulons have increased activity, but as nutrients become limited over time (8–10 hours) we noticed that the activities begin to come back to the reference state at 2 hours of growth. Another clear example of this dichotomy is observed when AB5075 is treated with azithromycin (Fig. 5c). Divergence of iModulon activities was observed to proceed in the opposite direction of the growth over the time data set, as increased azithromycin concentrations correlated to a dose-dependent increase in stress-associated iModulon activities and decrease in virulence-associated iModulon activities.

### iModulons highlight the importance of large plasmid p1AB5075 in pathogenesis

**Fig 6:**
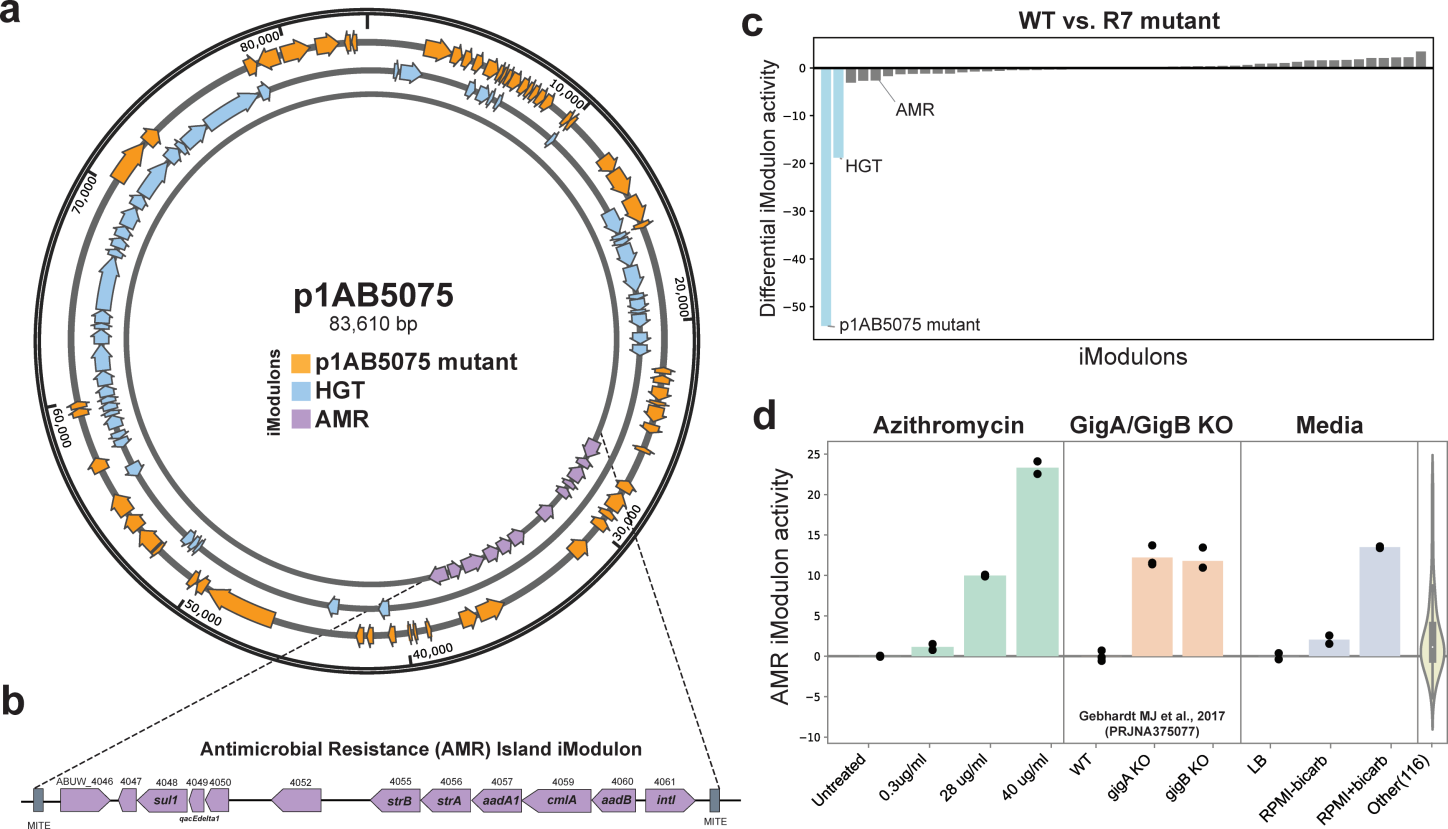
p1AB5075 plasmid-associated iModulons. (**a**) p1AB5075 plasmid map identifying the distribution of the genes within three plasmid-associated iModulons; p1AB5075 mutant, horizontal gene transfer (HGT), and antimicrobial resistance (AMR) iModulons. (**b**) The AMR iModulon captures only the genes within the plasmid-borne AMR island flanked by MITE sequences. (**c**) Differential iModulon activity (DIMA) analysis of all iModulons in R7, a mutant of AB5075 that lacks the p1AB5075 plasmid, relative to the wild-type AB5075. Note that values are normalized to <ref cond>, which has various activities for the three plasmid iModulons resulting in different apparent activities after the deletion; the AMR iModulon exhibits little activity in the reference. (**d**) AMR iModulon activity in azithromycin treatment, *gigA*/*gigB* knockouts, and growth in different media conditions: LB, RPMI +10% LB with bicarbonate, and RPMI +10% LB without bicarbonate.

*A. baumannii* AB5075 harbors three plasmids; the largest of these, p1AB5075, is ~83.6 Kb in size. p1AB5075 is of clinical importance because it confers resistance to aminoglycosides, chloramphenicol, and sulfonamides and can mediate the spread of resistance to other bacterial populations *via* conjugation. Three *Abaum*PRECISE iModulons (p1AB5075 mutant, HGT, and AMR) were found to be associated with this plasmid. Each of these three iModulons captures slightly different groups of genes on the plasmid (Fig. 6a). The p1AB5075 mutant iModulon is the largest of the three and shares overlap with each of the other two, whereas there are no common genes between the HGT and AMR iModulons (Fig S5). The HGT (**H**orizontal **G**ene **T**ransfer) iModulon is the only iModulon that includes the complete *tra* locus that encodes the conjugation apparatus necessary for plasmid spread in addition to three transposase genes (*tniABC*) located on the genome. The AMR (**A**nti**M**icrobial **R**esistance) iModulon captures the complete antibiotic resistance island spanning approximately 12 kb of p1AB5075 and flanked by miniature inverted-repeat transposable elements (MITE) (Fig. 6b).

Unlike the other two iModulons, the AMR iModulon had differential iModulon activity in a number of conditions, but not in an AB5075 strain lacking the p1AB5075 plasmid (R7 strain) relative to the wild type (Fig. 6c; Fig S6). As ICA extracts independent signals from mixed transcriptomic and genetic variations, this finding suggests that regulation of the antibiotic resistance island plays a role in conditions independent of simple plasmid loss. It is possible that this iModulon captures the effects of a previously described sRNA responsible for an opacity phase switch (51). AMR iModulon activities increased in the presence of antibiotic stress (azithromycin treatment) and decreased in *gigA* and *gigB* knockout mutants that lack a functional virulence-associated GigA-GigB TCS (Fig. 6d). Prior studies have shown that the GigA-GigB axis plays a role in the regulation of genes required for stress response during antibiotic exposure (40). Growth in different media conditions changes bacterial physiology, and we noted that AMR iModulon activity increased under host-representative tissue culture-type media conditions (RPMI +10% LB) compared to pure bacteriological media (LB). The increase in AMR iModulon activity reverted when AB5075 was grown in physiological media (RPMI +10% LB) lacking bicarbonate, suggesting bicarbonate may serve as a biological cue for adaptive antibiotic resistance (Fig. 6d).

## DISCUSSION

Deciphering the global TRN of emerging pathogens can provide new insights and guide hypothesis development regarding the complex mechanisms of their pathogenesis. From this perspective, *A. baumannii* remains understudied in comparison to other bacterial pathogens. To expand the knowledge base on the *A. baumannii* TRN, we used a blind source separation algorithm, ICA, to extract independent signals from *AbaumPRECISE (Acinetobacter baumannii* Precision RNA-seq Expression Compendium for Independent Signal Extraction), a curated compendium of 139 high-quality RNA-seq experiments. In this manner, 49 independently modulated gene sets, or iModulons, were generated with 36 assigned to biologically relevant regulators and/or functions. Analyzing the gene weights within the iModulons and their activities across *AbaumPRECISE* experimental conditions, we began to showcase how this model may help address specific concerns of the scientific, clinical, or public health communities. For example, analysis of iModulons informed organism-specific stress response mechanisms, stress-virulence trade-offs that might guide approaches to limit pathogenicity and identification of conditions that may induce antibiotic resistance.

As a whole, the *A. baumannii* iModulon structure appears virulence centric. While we categorized iModulons based on function, certain categories have meaningful overlap. For example, the regulation of iron (52, 53), manganese (54), phosphate (55), potassium (56), and sulfur (57) captured by the inorganic ion iModulons all are known to play an important role in bacterial evasion of host-mediated nutritional immunity, thereby holding implications for virulence. Following examples like these, one may propose that a large portion of the *A. baumannii* TRN can influence virulence within the host and survival outside of it. By comparison, published iModulons in other organisms have reported higher proportions of iModulons associated with central metabolic pathways such as carbon, nitrogen, and purine/pyrimidine metabolism. These findings may include a sampling bias, as the experimental conditions within *Abaum*PRECISE reflect the general scientific community’s interests in this emerging pathogen, which are mostly focused on disease contexts. But ICA *per se* is an untargeted, and ostensibly unbiased, analytical tool, and it remains plausible that *A. baumannii* could prioritize regulation of virulence/survival-promoting pathways to a greater extent than other bacteria, which might enhance its superbug potential. Either way, the iModulon structure provides a concise review of the current state of *A. baumannii* research.

Bacteria re-allocate cellular resources between tasks based on immediate need. Sometimes, this economization of energy takes the form of a trade-off where one trait is sacrificed for another that is more advantageous in the defined condition. Overexpression of *rpoS* in *E. coli* correlates with increased resistance to environmental stress but at the expense of metabolic capabilities (58, 59). Previous iModulon studies in *E. coli* and *S. aureus* have highlighted fear (RpoS/SigS iModulons) versus greed (Translation iModulons) trade-offs (19, 20). In our *A. baumannii* ICA model, we see this as a stress versus virulence trade-off in the form of an activity cluster encompassing the five iModulons with the highest explained variance. This activity cluster may represent global regulatory stress versus virulence trade-off, with GacA-GacS and PaaX (virulence) versus ABUW_1645, Translation and ppGpp (stress). It is possible that higher-level regulators like BfmR-BfmS and DksA modulate this stress-virulence iModulon cluster, as recent experimental studies highlight the role of these global regulators in divergent stress protection regulation and virulence (60–63). It is important to note that stress and virulence are multifaceted but, in this example, we limit stress to conditions that induce a fear-based state seen in initial adaptation or nutrient depletion during lag or stationary phase growth. Similarly, virulence in this context refers only to resistance to host innate immunity as seen in VIR-O phenotypes. Other stress responses and virulence mechanisms in *A. baumannii* are accounted for by other iModulons excluded from this activity cluster.

Azithromycin, an antibiotic rarely used in the treatment of Gram-negative bacterial infections due to its unfavorable potency in standard testing on bacteriological media, increases the susceptibility of *A. baumannii* to host defense peptides *in vitro* and innate immune clearance *in vivo* (64). We observed that azithromycin modulated a number of iModulons in our model, including the five iModulon virulence-stress clusters in a dose-dependent manner. Thus, one contributing factor to the unexpected efficacy of azithromycin against *A. baumannii* in synergy with host defenses is transcriptional regulatory dampening of virulence to prioritize survival against the antibiotic. Although azithromycin treatment shifted the stress-virulence dichotomy toward the avirulent phenotype, adaptive resistance to antibiotics may be part of the stress response seen in increased activity of AMR iModulon genes associated with p1AB5075 plasmid. Optimal antibiotic regimens to blunt pathogen virulence may thus require combinatorial approaches (65), to best target the pathogen based on their range on the stress-virulence spectrum.

*Abaum*PRECISE incorporates a number of data sets from different labs across the world. Mixed opaque (VIR-O), translucent (AV-T), and capsule positive/negative variants can be selected when *A. baumannii* cultures are left in a stationary phase for extended periods (41, 42, 66, 67) and if samples are not processed from purified AB5075 variants, the above phenomenon can pose a significant hurdle for accurate analysis of RNA-seq differential gene expression. However, as ICA can separate independent source signals from a mixed signal, it is possible for ICA to distinguish signals from these variants, and in theory, this should allow for accurate interpretation of our data. This utility of ICA decomposition is seen in the case of the ABUW_1645 iModulon, which captures the differential iModulon activity between purified VIR-O and AV-T variant populations. However, there is currently a lack of capsule-related iModulon or conditions that would capture transcriptional circuitry behind capsule variants. Another point of concern is in the labeling of the GacA-GacS iModulon, which was based on correlating PaaX iModulon activities in addition to differential iModulon activity in *gacA*::T26. Transposon insertion can result in polar effects on downstream adjacent genes out of the direct regulon, for example, transposon insertion in *vacJ* may modulate the expression of the immediately downstream *gigA* and *gigB* genes. These points present limitations of this study, as well as the field in general, since pooling published data in this way inherently exposes a shortage of ideal controls.

iModulon analysis also frames new questions amenable to further analysis by molecular and genetic means. GacA-GacS and ABUW_1645 iModulons harbor four genes in common but these genes are annotated as hypothetical proteins with unknown functions; targeted mutagenesis and heterologous expression studies may help decipher the full phenotypic impact of this potential RpoS-independent stress response. In addition, the iModulon activities we identified often mirror reported *A. baumannii* phenotypes in literature, but there are instances where this was not the case. For example, the directionality of AMR iModulon activity for *gigA* and *gigB* knockout mutants contradicts previous findings that these mutants in AB5075 have increased sensitivity to kanamycin and gentamicin (40). There are multiple mechanisms for aminoglycoside resistance in *A. baumannii* (i.e., RND efflux pump systems and aminoglycoside modification), and it is possible that GigA-GigB positively regulates a single mechanism, and *gigA* or *gigB* knockouts could have compensatory mechanisms to resist aminoglycoside stress, such as upregulation of the p1AB5075 AMR island encoding aminoglycoside modification mechanisms. Testing the susceptibilities of double knockouts of *gigA*/*gigB* and p1AB5075 genes (such as *aadB*) may tell us the contributions of each of these resistance mechanisms systems as parts of the whole phenotype.

Even with our detailed characterization of the *A. baumannii* iModulon structure, there still remain 13 iModulons that are uncharacterized. This could be due to the large number of unannotated genes within the *A. baumannii* genome. Probing the role of these gene sets will further the construction of a complete TRN in *A. baumannii*. Mechanistic insights into the intricate transcriptional regulatory network at play within this pathogen may provide evidence to inform treatment practices to guide bacterial populations to reduced fitness phenotypes for an increased likelihood of positive treatment outcomes.

## MATERIALS AND METHODS

### Bacterial sample preparation for RNA extraction and library preparation

For experiments assessing growth over time, an overnight culture of AB5075 was inoculated in physiological media (RPMI + 10% LB) and samples were collected at 2-hour time points for 10 hours. For comparison of mutants to wild type, AB5075 and mutant strains were grown to mid-exponential growth phase (OD_600_ = ~0.4). For assessing differences between antibiotic-treated and -untreated samples, mid-exponential growth phase cultures were exposed to varying concentrations of the antibiotic for 15 minutes prior to sample collection. For testing variations from culturing conditions, AB5075 was grown in a variety of culturing conditions (CA-MHB, LB, RPMI +10% LB, M9) with additional adjustments to parameters such as pH and supplementation of amino acids and bicarbonate prior to collection after 2 hours. All samples were prepared and collected in biological duplicates. Immediately at the time of sample collection, 3 mL of culture was added to 6 mL of Qiagen RNA-protect Bacteria Reagent, vortexed for 5 seconds, and incubated at room temperature for 5 minutes. After centrifugation, the supernatant was decanted, and the cell pellet was stored at −80°C. RNA was extracted using the Zymo Research Quick-RNA MicroPrep Kit per vendor protocol. An on-column DNase treatment was performed for 30 minutes at room temperature. The ribosomal RNA was removed using anti-rRNA DNA Oligo mix and Hybridase Thermostable RNase H (Biosearch Technologies, H39500) as previously described (68). A Swift RNA Library Kit was used following the manufacturer’s protocol to create sequencing libraries.

### Collection of publicly available RNA-seq metadata

In addition to the RNA-seq experiments performed in the laboratory, we incorporated all publicly available metadata for *A. baumannii* international clonal complex CC1 strains AB5075, AYE, and AB0057 from SRA for the ICA model using script reported to uncover all RNA-seq data for *P. aeruginosa* (34) (https://github.com/avsastry/modulome-workflow/tree/main/1_download_metadata), on NCBI SRA as of 20 August 2020. We manually selected *A. baumannii* samples that were for AB5075, AYE, and AB0057.

### RNA-seq data processing

RNA-seq data were processed using the modulome workflow (34). The data were downloaded and aligned to the AB5075 genome and plasmids (GenBank Accession: CP008706.1, CP008707.1, CP008708.1, CP008709.1) using the pymodulon modulome-workflow package: https://github.com/avsastry/modulome-workflow.

### Curation of metadata to generate high-quality compendium: *Abaum*PRECISE

All metadata generated through this work and publicly available from SRA were subject to a quality control pipeline, as previously described (34). Samples that failed FastQC had less than 500,000 reads mapped to coding sequences or had poor correlations with other data sets or within replicates (Pearson R value <0.9) were discarded. Samples that passed were compiled as *Abaum*PRECISE. Log-TPM data (**Data Set S2**) were generated for each BioProject normalizing to a project-specific reference condition detailed under “reference condition” in **Data Set S1**

### Computing and characterizing iModulons

iModulons were computed as previously described (19). Dimensionality was selected at 120 to optimize for a stable decomposition structure maximizing robust components and minimizing single gene components. The dimensionality was determined using OptICA. To enrich iModulons with known regulators and prophage regions, we first compiled a TRN/prophage data set based on various inputs: bidirectional best hits in AB5075 for RegPrecise data for strain AB0057, PHASTER-defined prophage regions in AB5075, and manually annotated regulon information from the literature. Enrichment of each iModulon against this data set was computed using Fisher’s Exact Test with the false discovery rate (FDR) of 10^−5^. Subsequent characterization was done by comparison of iModulons to those in *E. coli* (35) and *P. aeruginosa* (25). This was done using the functions within pymodulon.compare (34). First, bidirectional best hit (BBH) orthology between AB5075 and *E. coli* K-12 MG1655 and *P. aeruginosa* PAO1 was generated using BLAST (38). Using the compare_ica command, gene weights of *A. baumannii* iModulons were compared to ortholog gene weights in iModulons of *E. coli* and *P. aeruginosa*. Final characterization was done by manual annotation of the gene sets based on gene and COG annotations. Each iModulon was also categorized based on function (virulence, inorganic ions, energy metabolism, stress, functional, amino acid metabolism, and carbon metabolism) or associations with manipulations of the genome or with a plasmid.

### Calculating differential iModulon activity and activity clusters

Differential iModulon activity analysis and clustering were performed as described previously (19, 23, 27). Briefly, a log-normal distribution was fit to the difference of iModulon activities in biological replicates for each iModulon. Comparison of iModulon activity between two conditions was performed by calculating the absolute value of the difference of the average activity and then comparing it to the log-normal distribution using a two-tailed test to determine significance. 1-D Differential iModulon Activity (DIMA) bar plots were constructed by comparing iModulon activities between two conditions, normalizing reference conditions to 0. iModulon activity clustering was done using a Seaborn clustermap, then the scikit-learn agglomerative clustering function was applied and Spearman R correlation was calculated to identify iModulons with correlated activities across all conditions.

## Data Availability

Generated RNA-seq data are uploaded in NCBI SRA BioProjects PRJNA876061 and PRJNA658638. Details on all transcriptomic data sets used for the compilation of AbaumPRECISE and the normalized log TPM values for these data sets are listed in Supplementary Data Sets 1 and 2. With Supplementary Data Set 2 representing the X matrix, the M and A matrices are provided as Supplementary Data Sets 3 and 4, respectively. iModulon table with a summary for each iModulon is included in Supplementary Data Set 5. Further analysis of the contents of each iModulon and their activities can be accessed through the interactive AbaumPRECISE iModulon dashboard available at www.iModulonDB.org. The code to recreate this pipeline is available at https://github.com/SBRG/modulome_abaum.

## SUPPLEMENTAL MATERIAL

Data Set S1 msystems.00606-23-s0001.csv Details of *Abaum*PRECISE transcriptomic data sets.

Data Set S2 msystems.00606-23-s0002.csv X matrix (normalized log TPM values for *Abaum*PRECISE transcriptomic data sets).

Data Set S3 msystems.00606-23-s0003.csv M matrix.

Data Set S4 msystems.00606-23-s0004.csv A matrix.

Data Set S5 msystems.00606-23-s0005.csv All 49 iModulons with their function, evidence for characterization, category, gene count, and explained variance.

Supplemental material msystems.00606-23-s0006.pdf Figures S1-S6; captions for Data Sets S1 to S5.

ASM does not own the copyrights to Supplemental Material that may be linked to, or accessed through, an article. The authors have granted ASM a non-exclusive, world-wide license to publish the Supplemental Material files. Please contact the corresponding author directly for reuse.
