# Supplementary material for: Independent component analysis reveals 49 independently modulated gene sets within the global transcriptional regulatory architecture of multidrug-resistant Acinetobacter baumannii
Source: mSystems. 2024 Jan 8;9(2):e00606-23. doi: 10.1128/msystems.00606-23 (PMC10878099; doi:10.1128/msystems.00606-23)
Supplement: Supplemental material — Figures S1-S6; captions for Data Sets S1 to S5. [file msystems.00606-23-s0006.pdf]

Supplemental Materials

Independent component analysis reveals 49 independently modulated gene sets within the global transcriptional regulatory architecture of multidrug-resistant *Acinetobacter baumannii*

Nitasha D. Menon[1,2], Saugat Poudel[3], Anand V. Sastry[3], Kevin Rychel[3], Richard Szubin[3], Nicholas Dillon[2,4], Hannah Tsunemoto[5], Yujiro Hirose[2,6], Bipin G. Nair[1], Geetha B. Kumar[1], Bernhard O. Palsson[3], Victor Nizet[2,7]

[1]School of Biotechnology, Amrita Vishwa Vidyapeetham, Amritapuri, Kerala, India; [2]Division of Host-Microbe Systems and Therapeutics, Department of Pediatrics, University of California, San Diego, La Jolla, California, USA; [3]Department of Bioengineering, University of California San Diego, La Jolla, CA, USA; [4]Department of Biological Sciences, University of Texas at Dallas, Dallas, TX, USA; [5]Division of Biological Sciences, University of California, San Diego, La Jolla, California, USA, [6]Department of Microbiology, Graduate School of Dentistry, Osaka University, Suita, Osaka, Japan; [7]Skaggs School of Pharmacy and Pharmaceutical Sciences, UC San Diego, La Jolla, CA, USA.

Contents

Figure S1: iModulon gene weight plots comparing RpoH in *A. baumannii* with RpoH in *E. coli* and in *P. aeruginosa*.
Figure S2: iModulon gene weight plot comparing GacA-GacS and PaaX iModulons
Figure S3: Clustermap activity analysis of all *A. baumannii* iModulons.
Figure S4: Gene weights of the Translation iModulon and ppGpp iModulon
Figure S5: Comparative gene weight plots of the three p1AB5075 plasmid associated iModulons
Figure S6: p1AB5075 mutant and HGT iModulon activities across conditions of *Abaum*PRECISE

Supplemental Data Sets

Data Set S1: Details of *Abaum*PRECISE transcriptomic datasets.
Data Set S2: X Matrix (Normalized Log TPM values for *Abaum*PRECISE datasets).
Data Set S3: M matrix
Data Set S4: A matrix
Data Set S5: List of all 49 iModulons with their function, evidence for characterization, category, gene count, and explained variance.

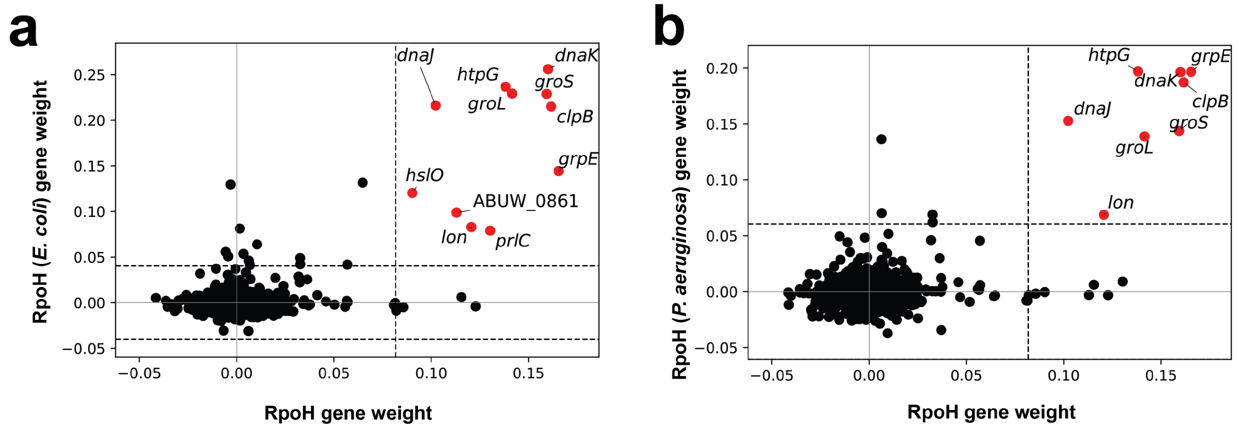

Figure S1: iModulon gene weight plots comparing RpoH in *A. baumannii* with RpoH in *E. coli* (a) and in *P. aeruginosa* (b). The red datapoints in comparative gene weight plots highlight genes that are common to both compared iModulons.

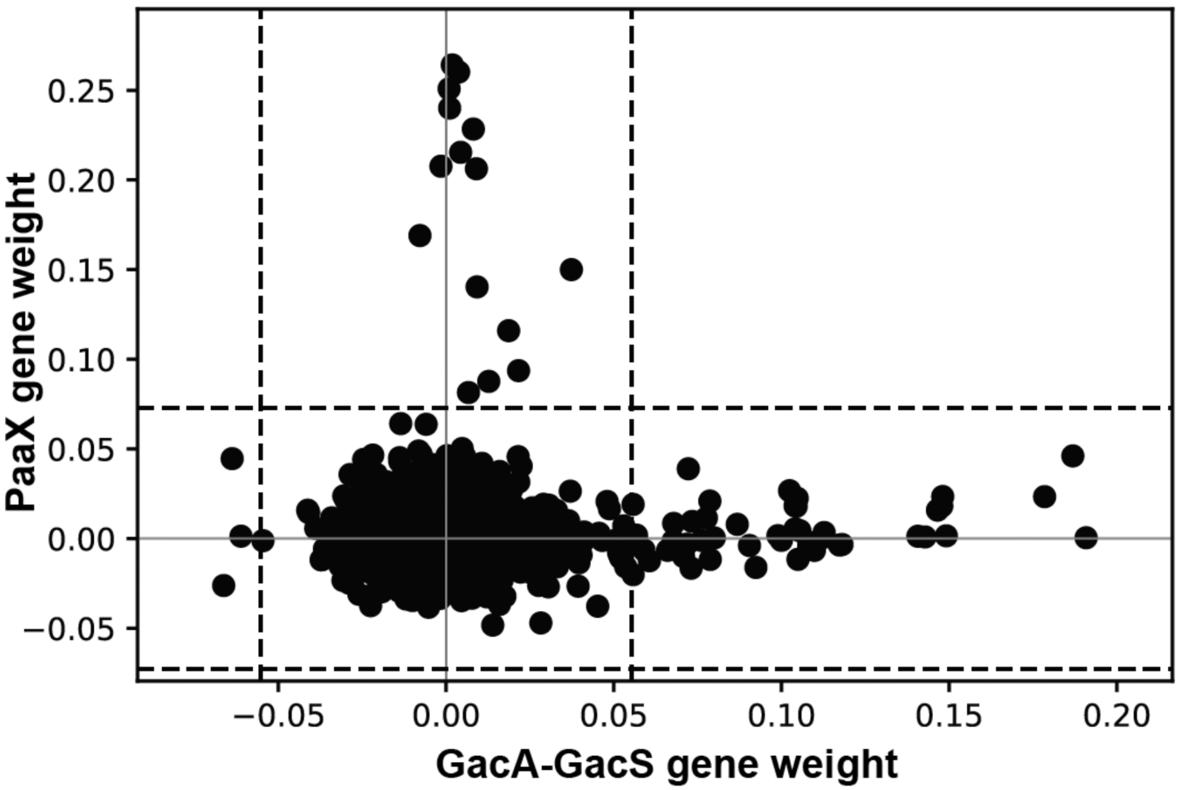

Figure S2: iModulon gene weight plot comparing GacA-GacS and PaaX iModulons.

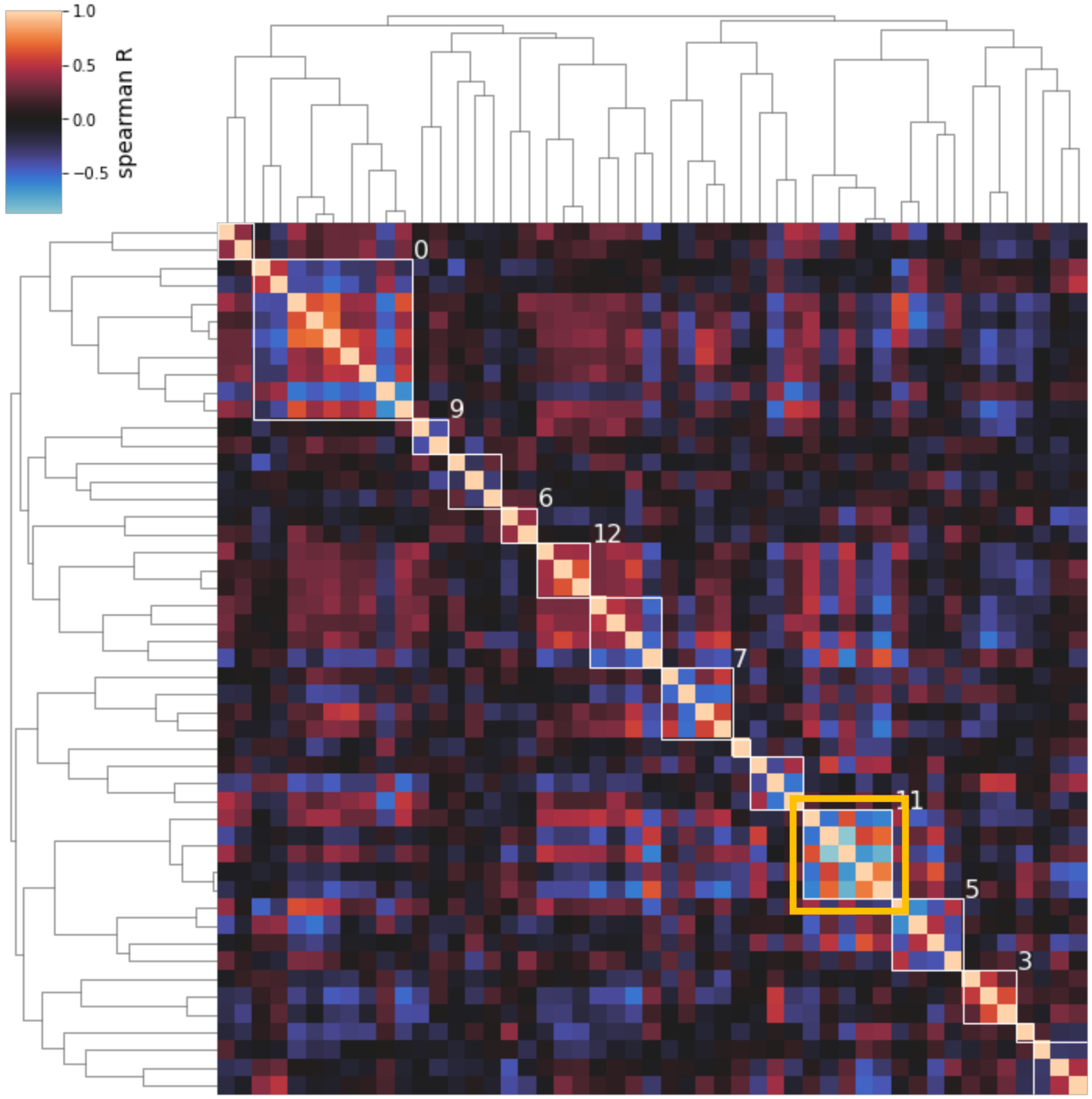

Figure S3: Clustermap activity analysis of all *A. baumannii* iModulons. Cluster 11 (highlighted) correlates to the stress-virulence cluster.

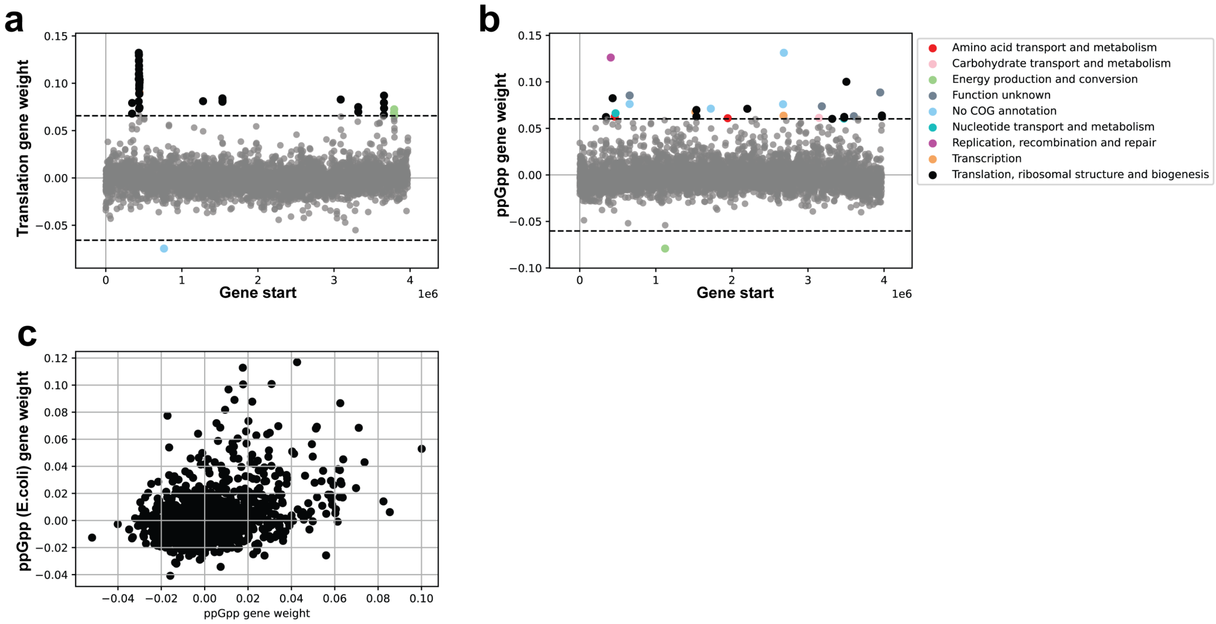

Figure S4: Gene weights of the Translation iModulon (a) and ppGpp iModulon (b). Comparative gene weight plots comparing ppGpp iModulons between *A. baumannii* and *E. coli* (c). The red datapoints in comparative gene weight plots highlight genes that are common to both compared iModulons.

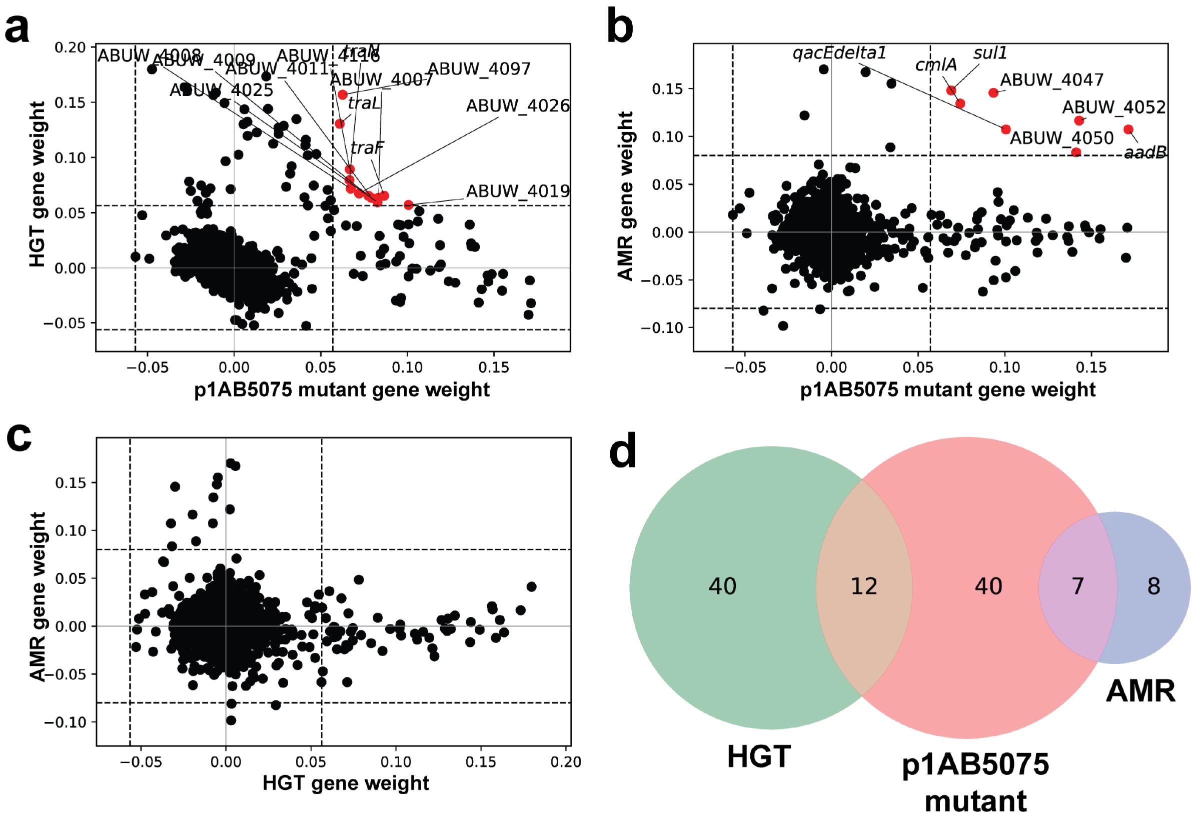

Figure S5: Comparative gene weight plots of the three p1AB5075 plasmid associated iModulons: comparing gene weights between p1AB5075 mutant and HGT (a), p1AB5075 mutant and AMR (b), and HGT and AMR iModulons. Venn diagram showcasing the gene overlaps between the iModulons (d). The red datapoints in comparative gene weight plots highlight genes that are common to both compared iModulons.

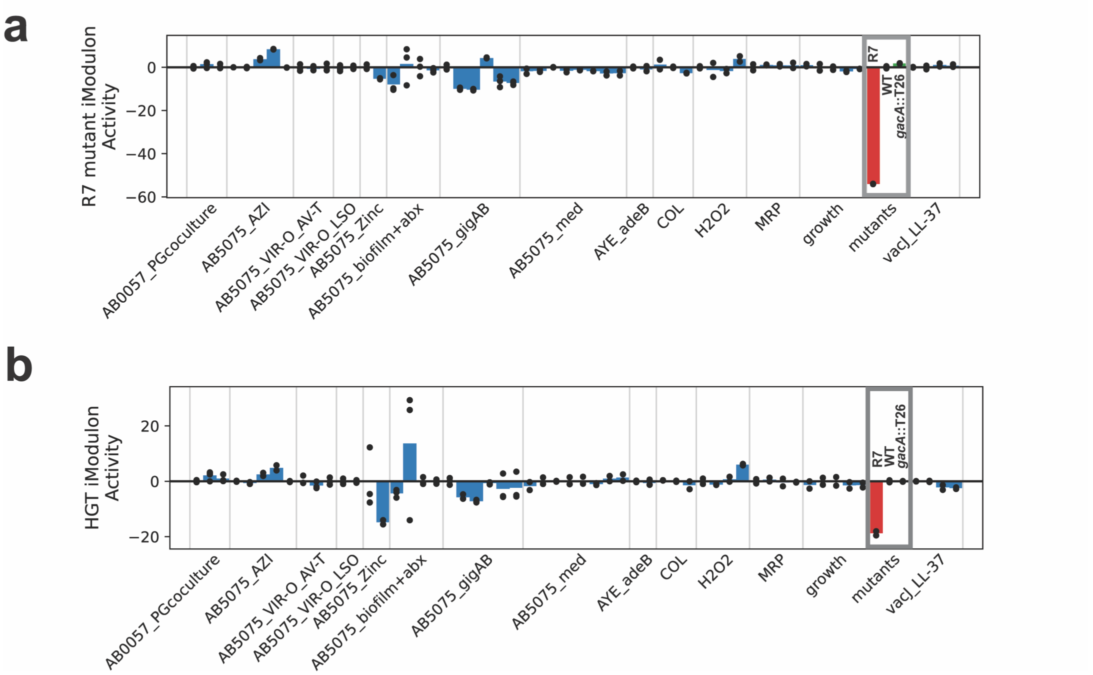

Figure S6: p1AB5075 mutant (a) and HGT iModulon (b) activities across conditions of *Abaum*PRECISE.
